# Macrophages enhance sodium channel expression in cardiomyocytes

**DOI:** 10.1007/s00395-024-01084-8

**Published:** 2024-10-09

**Authors:** N. V. Bogert, M. Therre, S. Din, J. Furkel, X. Zhou, I. El-Battrawy, J. Heineke, P. A. Schweizer, I. Akin, H. A. Katus, N. Frey, F. Leuschner, M. H. Konstandin

**Affiliations:** 1 Department of Cardiology, University Hospital Heidelberg, Ruprecht-Karls-University Heidelberg, Im Neuenheimer Feld 410, 69120 Heidelberg, Germany; 2 Department of Cardiology, University Medical Centre Mannheim, Ruprecht-Karls-University Heidelberg, Mannheim, Germany; 3 Department of Cardiology, BG Universitätsklinikum Bergmannsheil Bochum, Ruhr-University, Bochum, Germany; 4 Institut Für Forschung Und Lehre (IFL), Molecular and Experimental Cardiology, Ruhr University Bochum, Bochum, Germany; 5 Department of Cardiovascular Physiology, European Center for Angioscience, Medical Faculty Mannheim, Ruprecht-Karls-University Heidelberg, Mannheim, Germany; 6 German Centre for Cardiovascular Research (DZHK), Partner Site Heidelberg, Mannheim, Germany

**Keywords:** Macrophages, AV-node, AV-conduction, Cx43, Nav1.5, Sodium channel, Connexins

## Abstract

Cardiac macrophages facilitate electrical conduction through the atrioventricular-node (AV) in mice. A possible role for cardiomyocyte-macrophage coupling on the effect of antiarrhythmic therapy has not been investigated yet. Holter monitoring was conducted in LysM^Cre^xCsf1r^LsL−DTR^ mice (MM^DTR^) under baseline conditions and after an elctrophysiological stress test by flecainide. In vivo effects were recapitulated in vitro by patch-clamp experiments. The underlying mechanism was characterized by expression and localization analysis of connexin43 (Cx43) and voltage-gated-sodium-channel-5 (Na_v_1.5). ECG monitoring in MM^DTR^ mice did not show any significant conduction abnormalities but a significantly attenuated flecainide-induced extension of RR- and PP-intervals. Patch-clamp analysis revealed that the application of flecainide to neonatal rat ventricular cardiomyocytes (CMs) changed their resting-membrane-potential (RMP) to more negative potentials and decreased action-potential-duration (APD50). Coupling of macrophages to CMs significantly enhances the effects of flecainide, with a further reduction of the RMP and APD50, mediated by an upregulation of Cx43 and Na_v_1.5 surface expression. Macrophage depletion in mice does not correlate with cardiac electric conduction delay. Cardiac macrophages amplify the effects of flecainide on electrophysiological properties of cardiomyocytes in vivo and in vitro*.* Mechanistically, formation of macrophage-cardiomyocyte cell–cell-contacts via Cx43 facilitates the recruitment of Na_v_1.5 to the cell membrane increasing flecainide effects.

**Supplementary Information:**

The online version contains supplementary material available at 10.1007/s00395-024-01084-8.

## Introduction

Primitive macrophages seed the early embryonic heart by ∼E9.5. At this stage, they are yolk-sac-derived and long-lived [7]. The adult heart contains different macrophage subsets that can be differentiated through cell surface expression of the C–C motif chemokine receptor type 2 (CCR2) [2]. In both humans and mice, cardiac macrophages can be found not only dispersed throughout the myocardium but also in close proximity to the conduction system [13]. Seminal work has recently demonstrated the relevance of macrophages for electrical conduction in the heart [11, 26]. Patch-clamp analyses and computational modeling indicate that neighboring macrophages alter the electrophysiological properties of cardiomyocytes. Depletion of myeloid cells in CD11b^DTR^ mice resulted in 3^rd^-degree atrioventricular block (AV-block). Lack of macrophages in other genetic or pharmacological depletion strategies, however, resulted in milder conduction abnormalities [13].

Mechanistically, electrical coupling between cardiomyocytes and cardiac macrophages occurs via connexin43 (Cx43) gap junctions [8, 13, 21, 23]. The function of Cx43 as part of the gap junction reaches beyond its role as a pore molecule forming a direct cytoplasmatic connection between neighboring cardiomyocytes and thereby transmitting the action potential. Cx43 is also involved in the recruitment of the sodium channel Na_v_1.5, the main target of flecainide, to the cell membrane [22]. Flecainide is a class IC antiarrhythmic drug used in patients without structural heart disease suffering from paroxysmal supraventricular tachycardia (PSVTs), including atrioventricular nodal re-entrant tachycardia (AVNRT), AV re-entrant tachycardia (AVRT) and atrial fibrillation/atrial flutter.

In the herein study we analyzed the LysM^Cre^xCsf1R^LsL−DTR^ (MM^DTR^) mouse model [20], which enables a very selective depletion of monocytes and macrophages regarding electrophysiological conduction properties in the presence and absence of flecainide.

## Material and methods

### Animals

**Fig. 1:**
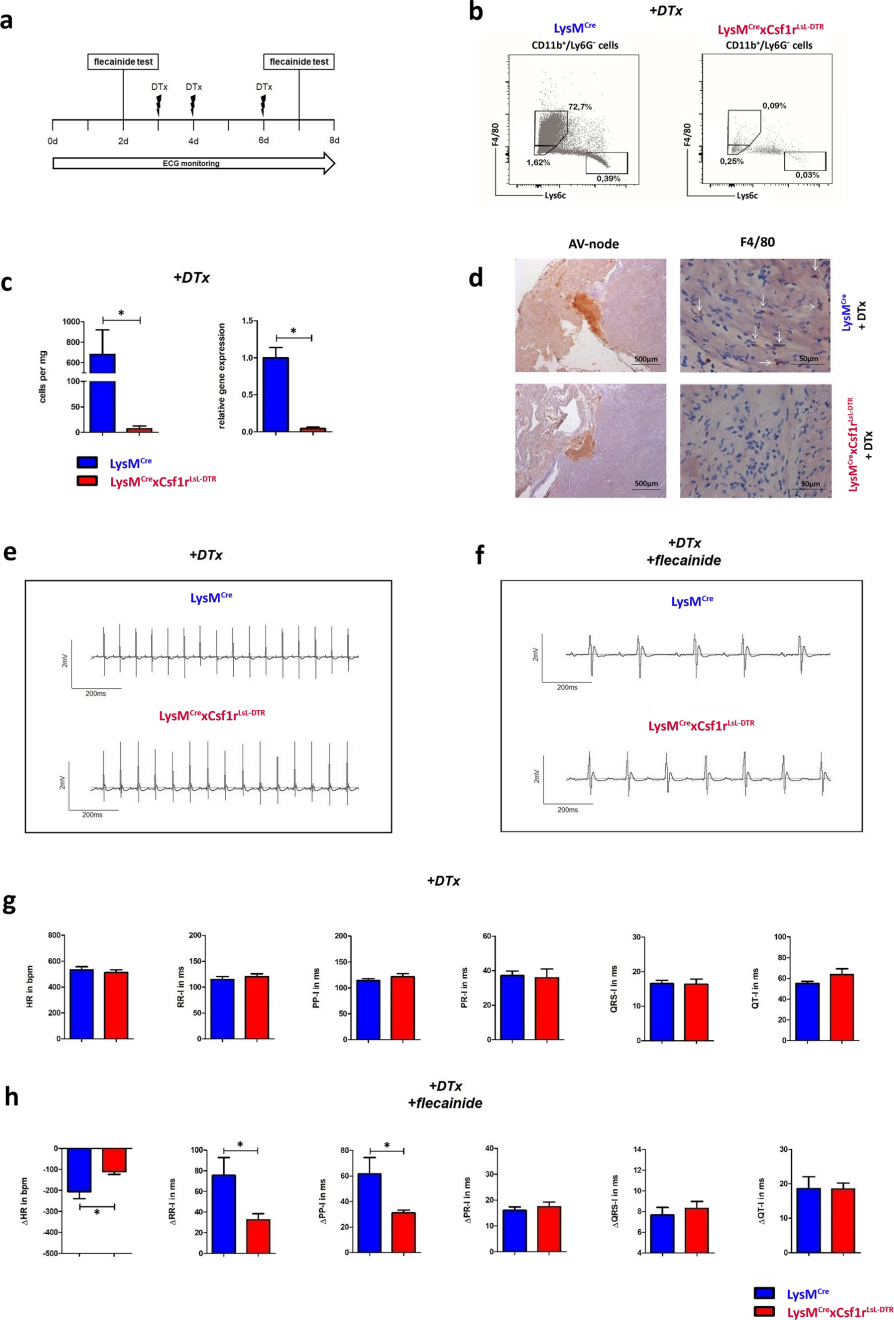
Depletion of macrophages in mice does not affect cardiac electrical conduction. LysM^Cre^ x Csf1r^LsL−DTR^ mice enable macrophage specific depletion in the heart by application of diphteria toxin (DTx). **(a)** ECG were recorded continuously and electrophysiological stress test was performed with flecainide. Myeloid depletion with DTx was started on day 3. **(b)** Heart samples were digested and analyzed by FACS of LysM^Cre^ (left, *n* = 4) and LysM^Cre^ x Csf1r^LsL−DTR^ mice (right, *n* = 4) after application of DTx. **(c)** Quantification of the heart samples by FACS (left) and qPCR (right) show a significant depletion of cardiac macrophages. **(d)** F4/80^+^ macrophages located in the AV-node are marked with white arrows. AV-nodes were detected by Koelle-staining and depletion of macrophages could be seen after DTx application in the AV-node as well. **(e–h)** After implantation of LT-ECG holter, electrical conduction was monitored prior and during the macrophages depletion period (*n* = 4). To further stress the cardiac electrical conduction system, we applied the sodium-channel inhibitor flecainide to unmask a higher AV-node vulnerability towards a reduced electrical conduction. **(e/g)** During macrophages depletion no significant changes could be detected comparing the electrical conduction times prior and during DTx application. **(f/h)** Even after the application of flecainide, no impaired electrical conduction towards the development of an AV-block could be detected. Nevertheless, we detected an impaired effect of flecainide in the absence of cardiac macrophages compared to control animals resulting in a significant lower decrease in heart rate (HR), PP-interval and RR-interval after flecainide application. *p < 0.05, data are shown as mean ± SD

All conducted animal experiments of this study were reviewed and approved by the Regierungspräsidium Karlsruhe (G246/14). For effective macrophage depletion analysis, mice expressing Cre under control of lysin motif (LysM) and diphteria toxin receptor (DTR) under control of colony stimulating factor 1 receptor (Csf1r) were used (Suppl. Figure 1a). Application of diphteria toxin (DTx) leads to a DTR mediated cell death in LysM and Csf1r double positive cells and causes a cell type specific depletion. Previous analyses showed that macrophages and monocytes are affected by this depletion but not dendritic cells [20]. For the initiation of myeloid depletion, 20 ng/kg bodyweight (BW) DTx was applied intraperitoneal, followed by an injection of 4 ng/kg BW DTx on the following day. To maintain myeloid depletion, doses of 4 ng/kg BW DTx were injected every second day. Electrocardiogram (ECG) holter monitors (ETA-F10, DataSci) were implanted under isoflurane anesthesia and electrodes were fixed on the pectoral muscles. Mice (LysM^Cre^xCsf1r^LsL−DTR^, *n* = 4; LysM^Cre^, *n* = 4) were monitored for 24 h (h) over the whole experimental protocol as illustrated in Fig. 1a. Flecainide was applied intraperitoneal adapted on the bodyweight (20 mg/kg BW) to demask potential cardiac atrioventricular conduction abnormalities. Recordings of the documented ECG were analyzed with Ponemah 5.2 (DataSci).

### Flow cytometry

Murine heart, spleen and blood were harvested and analyzed for myeloid subpopulations by flow cytometry. Hearts were digested with an enzyme-based digestion solution containing collagenase I (Sigma), collagenase XI (Sigma-Aldrich), hyaluronidase (Sigma-Aldrich), DNAse (Sigma-Aldrich) and HEPES (Gibco) to obtain a single cell suspension. Spleen samples were homogenized by passing through a 70 µm nylon filter (Greiner bio-one). Red blood cells were lysed with red blood cell lysis buffer (RBC lysis buffer) prior to the staining procedure and unspecific binding was reduced by Fc-block (BD Biosciences) application. Samples were stained with antibodies for 60 min (min) at 4 °C and myeloid subpopulations were analyzed after washing in flow cytometry buffer (2 mM ethylenediaminetetraacetic acid (EDTA) (sc-203932, Santa Cruz) and 2% fetal calf serum (FCS) (Gibco) in phosphate-buffered saline (PBS) by flow cytometry (FACS Verse, BD Biosciences). Analysis was performed using FlowJo 10 (BD Biosciences) and flow cytometry gating strategy is illustrated in Suppl. Figure 1e.

### Immunohistochemistry

Mouse hearts were washed in ice-cold Phosphate buffered saline (Sigma-Aldrich), embedded in Tissue-Tek^®^ O.C.T.™ Compound (Sakura) and snap-frozen in liquid nitrogen. Frozen sections were cut in slices of 6 µm thickness with a cryostat (SME, Thermo Shandon). Immunohistochemical identification of atrioventricular node tissue was assessed by Koelle-Staining as previously described [16]. For identification of macrophages, slides were fixed with acetone for 30 s (s) at -20 °C, followed by a blocking step with 20% donkey serum (Jackson ImmunoResearch) in Tris-buffered saline (TBS) for 1 h at room temperature and staining with F4/80 antibody 1:150 overnight at 4 °C. All appropriate washing steps were performed three times with 0,2% bovine serum albumin (BSA) (Aurion) in TBS. As a secondary antibody and for development, a biotin goat anti-rat Ig antibody (1:50), followed by a streptavidin-conjugated horseradish peroxidase (HRP) (1:400) and 3-amino-9-ethylcarbazole (AEC) substrate were applied for 15 min each. For Cx43- (1:400, 2 h room temperatue (RT)) and Na_v_1.5-staining (1:200, 2 h RT), slides were fixed with 2% paraformaldehyde (PFA) (Science-Services) for 10 min and secondary antibodies against the host species of the primary were labeled with alexa fluor (AF) 488 (1:400, 1 h RT). Finally, slides were mounted with an antifade mounting medium with 4′,6-diamidino-2-phenylindole (DAPI) (Vector laboratories). All slides were recorded with the same settings with a confocal microscope (SP8, Leica, Germany). Observers were blinded to the experimental group when scanning the slides as well as analyzing the data. A complete list of the used antibodies is provided in the supplemental material.

### Proximity ligation assay

Proximity ligation assay (PLA) was conducted according to the manufacturer`s recommendations (Duolink^®^ In Situ PLA^®^, Sigma-Aldrich). Briefly, frozen tissue sections as well as cells cultured on a chamber slide system (Thermo Scientific) were fixed with 2% PFA for 10 min, followed by three washes with PBS (Sigma-Aldrich). Next, sections were blocked with 0,5% Triton^™^ X-100 (Sigma-Aldrich) diluted in the kit`s blocking solution for 1 h at RT. Sections were then incubated with the primary antibodies anti-Na_v_1.5 (Alomone) and, depending on the experiment, anti-connexin43 (Invitrogen) or anti-pan-syntrophin (Invitrogen) diluted in the kit`s antibody diluent for 2 h at RT. The following steps were all performed in a humidified chamber at 37 °C with the appropriate washing steps according to the manufacturer`s protocol. After removal of the primary antibodies by washing 2 times in wash buffer A, the pair of PLA probes (a plus and a minus strand of oligonucleotides, conjugated to secondary antibodies against IgG of the primary antibodies` species) was applied for 1 h. The oligonucleotides were joined together by the enzyme ligase for 30 min, followed by an amplification step with the enzyme polymerase for 2 h. Fluorescently labeled nucleotides hybridize to the product of the amplification and were viewed with the same settings with a confocal microscope (Leica SP8) after mounting with the kit`s mounting medium with DAPI. Observers were blinded to the experimental group when scanning the slides as well as analyzing the data. For quantification, the mean fluorescence intensity (MFI) of the PLA signal was determined with AdobePhotoshop and normalized per field of view or normalized per cell, respectively.

### Cell culture, cardiomyocyte isolation, treatments

Neonatal rat ventricular cardiomyocytes (CMs) were isolated as previously described [9, 17]. After trypsin digestion, either 500,000 cells for RNA isolation or 1,000,000 cells for protein isolation were counted using a haemocytometer (Neubauer Improved Chamber) and plated per well. The wells have been pre-coated with 0,1% gelatin (Sigma) for 2 h at 37 °C. Cells were cultured with DMEM/F12 (Gibco) including 10% FCS (Gibco) and 1% Penicillin–Streptomycin (Gibco). For co-culture experiments, rat macrophages (NR8383 cell line, CRL-2192, ATCC) were added to CMs in a 1:1 ratio. Cells were incubated together at 37 °C and 5% CO_2_. After 48 h, macrophages were separated from CMs by 3 min of incubation with Accutase^®^ (Sigma) at 37 °C, followed by three washes with PBS. Successful separation was confirmed with light microscopy.

### Cellular electrophysiology

Co-culture of CMs and rat macrophages were carried out as described in the previous section. Electrophysiological characteristics of cardiomyocytes either without or with attached macrophages were analyzed after 24 h of seeding. Flecainide was applied at a concentration of 10 µM. In brief, patch-clamp glass pipettes were pulled from borosilicate glass capillaries (TW150F-4, world Precision Instruments) and were pulled with DMZ-Universal Puller (Zeitz-InstrumenteVertriebs GmbH, Martinsried, Germany) with tip resistances ranging from 3 to 4 MΩ after back-filling with internal solution (see below). All experiments were carried out at room temperature. Action potentials were evoked in current clamp mode with holding current of -40pA by injection of brief current pulses (2 ms, 1nA) at 0.2 Hz. Patch-clamp internal solution for current clamp action potential recordings was composed as follows (in mM): 10 HEPES, 126 KCl, 6 NaCl, 1.2 MgCl_2_, 5 EGTA, 11 glucose and 1 MgATP, pH 7.2 (KOH), and extracellular Tyrode’s solution consisted of 130 NaCl, 5.9 KCl, 2.4 CaCl_2_, 1.2 MgCl_2_, 11 glucose, 10 HEPES, pH 7.4 (NaOH). Functional coupling of macrophages and cardiomyocytes via gap junctions was examined with the Cell Meter^™^ Fluorescence Gap Junction Tracing Kit (AAT-Bioquest, Pleasanton, USA) according to the manufacturer manual (Suppl. Figure 2 h).

### Sample preparation, immunoblotting, qRT-PCR

Cells were lysed and RNA was enriched using the *Quick*-RNA Miniprep kit (Zymo Research) according to the manufacturer’s protocol. cDNA was transcribed using the iScript^™^ cDNA Synthesis Kit (Biorad) and a thermocycler (Mastercycler, Eppendorf). Real-time quantitative polymerase chain reaction was carried out with the iTaq^™^ Universal SYBR^®^ Green Supermix (Biorad) following the manufacturer’s protocol in a real-time PCR system (ViiA 7, Applied Biosystems). Differences were calculated with the ^ΔΔ^C(T) method. A complete list of the used primers and antibodies is provided in the supplemental material.

Briefly, mice were sacrificed, hearts removed, washed in ice-cold PBS and snap frozen in a tube in liquid nitrogen. Murine cardiac tissue was lysed by using a tissue homogenizer (Precellys^®^, VWR) in lysis buffer (20 mM TRIS hydrochloride (Carl Roth), 150 mM sodium chloride (Sigma-Aldrich), 1% Triton^™^ X-100 (Sigma-Aldrich), 0,1% sodium dodecylsulfate (Sigma), complete^™^ (Roche), PhosSTOP™ (Roche) and stainless steel beads. Homogenization was carried out with the tissue homogenizer Precellys®. If needed, homogenization was followed by RNA isolation with TRIzol^™^ (Invitrogen) using standard procedures. For Immunoblotting, protein samples of lysed tissue were loaded onto a 4–12% precast polyacrylamide gel (Biorad) for electrophoresis. Separated proteins were then transferred onto a polyvinylidene fluoride membrane (Merck Millipore), blocked with 5% skim milk in Tris-Buffered Saline and 0,1% Tween-20 (TBS-T) for 1 h at RT and exposed to primary antibodies overnight. Horseradish peroxidase (HRP) IgGs (Cell Signaling) were used as secondary antibodies for 1 h at RT. Signals were detected on a CL-XPosure^™^ Film (ThermoScientific) by using a development machine (Cawomat 2000 IR, Cawo).

### Statistics

Statistical analyses were conducted using GraphPad Prism 7 (GraphPad Software Inc). Data are shown as a bargraph or dot plot with mean ± SD. Gaussian distribution was analyzed by Kolmogorov–Smirnov test, followed by student`s *t*-test or Wilcoxon-Mann–Whitney-test accordingly. *P* values < 0.05 were considered significant.

## Results

### Depletion of macrophages in MM^DTR^ mice does not delay AV-conduction but enhances the effects of flecainide in vivo

While removal of myeloid cells in CD11b^DTR^ mice led to 3rd degree AV-blocks, other murine macrophage depletion strategies resulted in milder phenotypes. *Schreiber and colleagues* have previously established LysM^Cre^xCsf1R^LsL−DTR^ (MM^DTR^) mice, which enables the selective depletion of monocytes and macrophages, but sparing other myeloid cell subsets [20]. In this model, mice expressing Cre-recombinase under the LysM promotor are bred with animals expressing a floxed STOP codon upstream of the DTx-receptor under the Csf1r promotor. Therefore, only cells with activity in both promotors (Csf1r and LysM), namely macrophages and monocytes, will be sensitive to depletion by DTx-injection (Suppl. Figure 1a). We investigated these mice for the development of conduction disorders, littermates expressing LysM^Cre^ but not Csf1r^LsL−DTR^ served as controls. To dismantle possible undetected conduction system disorders, flecainide was given on day 2, one day before induction of macrophage depletion by DTx-injection on days 3, 4 and 6. At the time of macrophage depletion the flecainide provocation test was repeated on day 7. ECG was monitored continuously and Fig. 1a shows the experimental protocol. Successful depletion of cardiac macrophages was confirmed by flow cytometry (Fig. 1b) and quantification of F4/80^+^ absolute cell numbers is depicted in Fig. 1c on the left. The complete flow cytometry gating strategy is provided in Suppl. Figure 1e. qRT-PCR analysis of whole heart tissue also indicated robust macrophage depletion in our setup: F4/80 gene expression was significantly attenuated (Fig. 1c, right), as well as CD206 gene expression (Suppl. Figure 1d). Besides the depletion of macrophages, a reduction of monocytes was detected (Suppl. Figure 1b/c). Immunohistochemical analysis of AV-nodes, identified by Koelle-staining, showed F4/80^+^ cells in closest proximity to the cardiac conduction system (Fig. 1d) and robust depletion of macrophages in MM^DTR^ mice. Analysis of holter ECG recordings at baseline revealed no differences between controls and MM^DTR^ mice regarding heart rate, PP-, RR- PR- QRS- or QT intervals (Suppl. Figure 1f). Also, after the first flecainide provocation test before the first DTx injection (day 2), both strains did not show a significant difference in these time intervals or heart rate (Suppl. Figure 1 g). Unexpectedly, no conduction abnormality was detectable upon macrophage depletion by DTx-injection. Heart rate as well as PP-, RR- PR- QRS- or QT intervals did not show any significant difference as shown in Fig. 1e/g. Furthermore, flecainide provocation under conditions of macrophage depletion (day 7) did not demask any delay in AV-conduction or any form of AV-blockade (Fig. 1f/h). The PR-, QRS- and QT intervals were identical in both strains. However, the flecainide-induced deceleration of the heart rate and prolongation of the RR- as well as PP- interval was significantly attenuated in the MM^DTR^ mice. In summary, macrophage depletion in the MM^DTR^ mouse does not affect AV-conduction, however, the response towards flecainide in terms of heart rate reduction and RR- and PP interval extension were attenuated, suggesting that flecainide mediates its effects in vivo at least partially at the sinus node or sinoatrial conduction system via cardiac macrophages.

### Macrophages enhance the effects of flecainide on cardiomyocytes in vitro

**Fig. 2:**
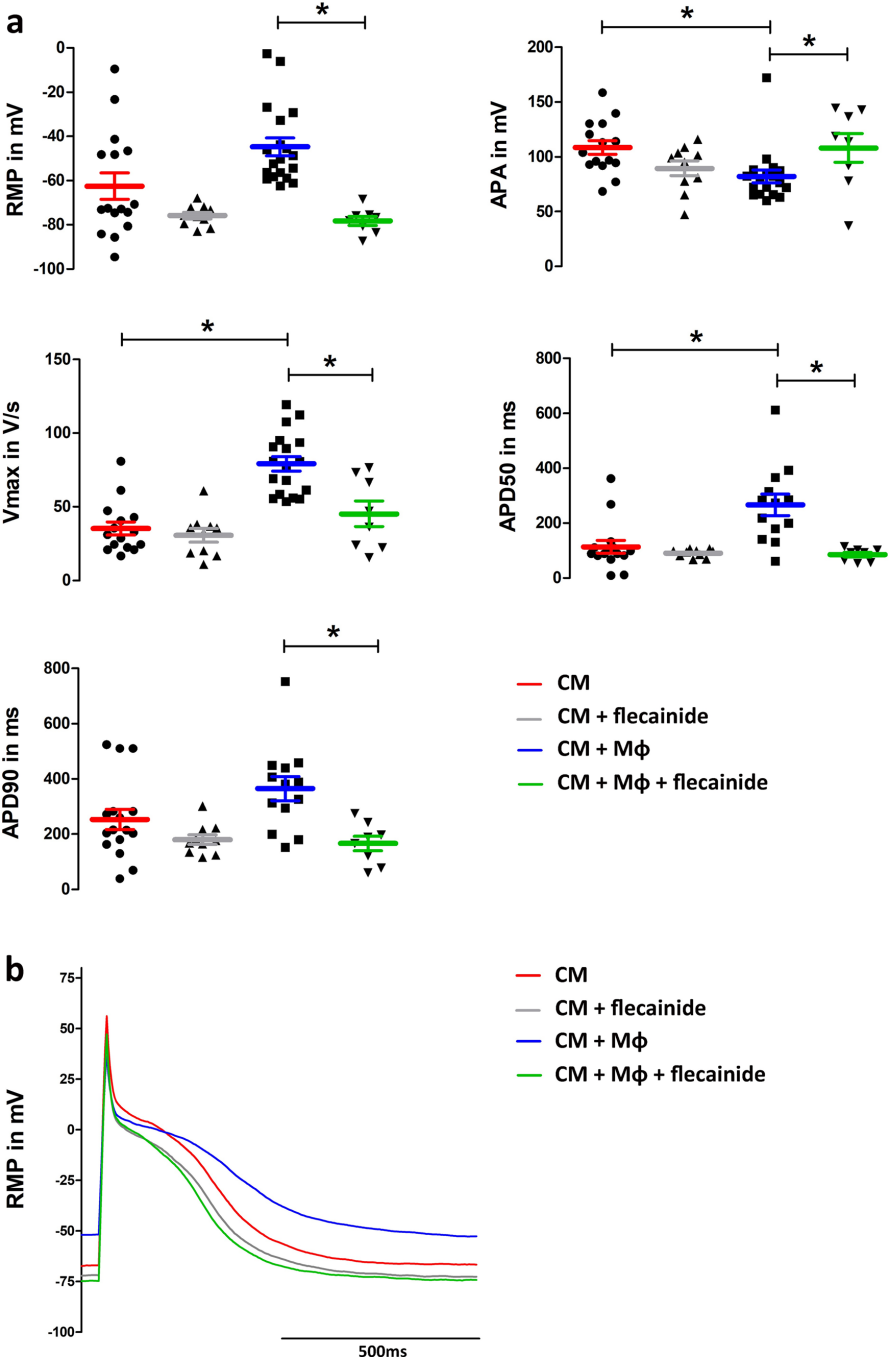
Macrophages enhance the effects of flecainide on cardiomyocytes. For electrophysiological analysis, patch-clamp of CMs in co-culture conditions with macrophages were compared with a single suspension of CMs. **(a)** Co-culture experiments show a more positive RMP and a decrease in APA with an increase in Vmax and APD50. Application of flecainide does not significantly change the absolute values in CM alone. However, we can see the expected change as a trend towards a lower V_max_ after the application of flecainide. In contrast, under co-culture conditions with macrophages significant changes can be observed upon flecainide treatment. Numbers of individual experiments are indicated in the figure. **(b)** Representative AP-traces of each experimental condition. *p < 0.05, data are shown as mean ± SD

Coculture experiments of NR8383 macrophages with CMs were conducted to evaluate the possible direct effects of macrophages upon cardiomyocytes. As expected, coupling of macrophages to cardiomyocytes caused a shift towards a more positive resting membrane potential (RMP), a decrease in action potential amplitude (APA) and a raise in maximum velocity of depolarization (Vmax) and as well as AP duration at 50% (APD50) in patch-clamp analyses (Fig. 2a). Application of flecainide in CMs leads to a more negative RMP, a decrease in APA and a decrease in APD50 and AP duration at 90% (APD90), while Vmax did not change significantly. Application of flecainide in CMs in the presence of macrophages leads to a significant change to a more negative RMP and a significantly stronger shortening of APD50 compared to CMs alone (Fig. 2a). Also, in the presence of macrophages the flecainide-induced increase in APA was significantly higher. This indicates that macrophages enhance the effects of flecainide on cardiomyocytes in vitro*.* Representative AP-traces are shown in Fig. 2b.

### Macrophages induce Na_v_1.5 cell surface expression in vivo and in vitro

**Fig. 3:**
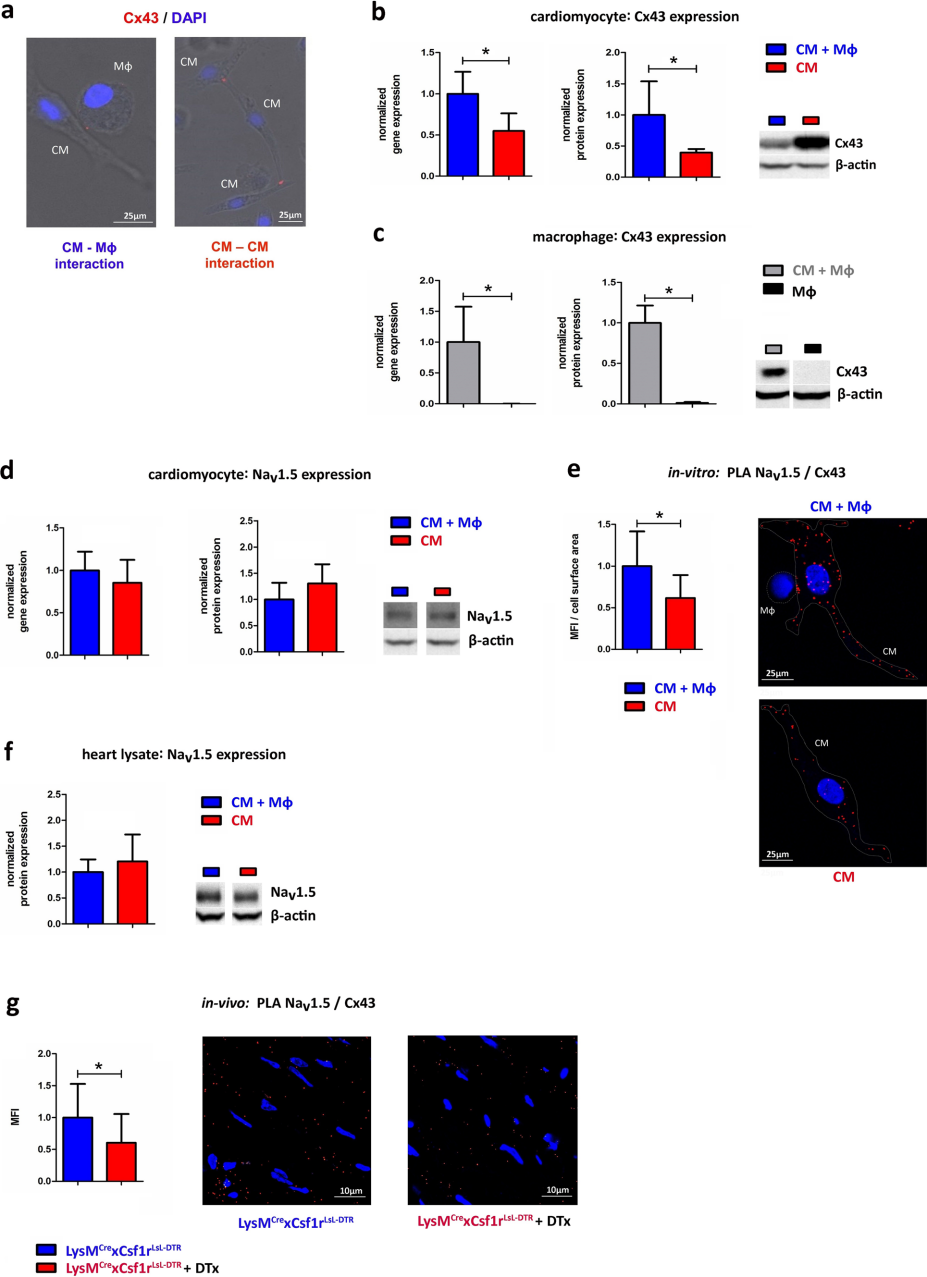
Macrophages regulate Na_v_1.5 expression in vivo and in vitro. (**a**) Co-culture of CMs with rat macrophages (Mφ, NR8383) leads to a Cx43 expression between cardiomyocytes and macrophages. **(b/c)** Cx43 is upregulated in cardiomyocytes and in macrophages on gene expression analyzed by qRT-PCR (left, *n* = 8) and total protein level analyzed by immunoblot (right, *n* = 8). **(d)** Co-culture experiments of CMs and macrophages show no significant change in total Na_v_1.5 gene and protein expression analyzed by qRT-PCR (left, *n* = 8) and immunoblot (right, *n* = 8). **(e)** Immunohistochemical analysis by PLA shows an increased surface expression of Na_v_1.5 under co-culture conditions (CM + Mφ *n* = 13, CM *n* = 10). **(f/g)** In vivo analysis of murine heart samples (*n* = 4) shows no significant change in the total protein amount of Na_v_1.5 but a significant decrease in Na_v_1.5 surface expression analyzed by PLA in the absence of cardiac macrophages. *p < 0.05, data are shown as mean ± SD

Direct contact of cardiomyocytes with macrophages via Cx43 attenuates their RMP [13]. Cx43 is also known to play a crucial role in regulating the trafficking of cardiac sodium channels Na_v_1.5 to the membrane [22]. Therefore, we first evaluated the presence of Cx43 between neighboring macrophages and cardiomyocytes in our cell culture conditions by immunohistochemistry (Fig. 3a), confirming that coupling between macrophages and cardiomyocytes occurs in our hands. Next, Cx43 expression was analyzed by qRT-PCR and immunoblot in cardiomyocytes and macrophages cultured either separately or after co-culture. In isolated macrophages no Cx43 was detectable, while cardiomyocytes alone do express Cx43 (Fig. 3b/c). After co-culture both cell types were isolated and Cx43 expression was re-analyzed. Co-culture induced Cx43 expression in CMs on mRNA and protein level by 1.82 fold and 2.53 fold, respectively. Macrophages isolated from coculture also expressed Cx43 on mRNA and protein level. Immunoblot raw data are provided in detail in Suppl. Figure 2a-d. Potential effects of secretory mediators could be excluded through treatment of the control group with macrophages’ supernatant (Suppl. Figure 2e). The efficacy of macrophage separation from cardiomyocytes after coculture was monitored by FACS analysis (Suppl. Figure 2 g).

Regarding Na_v_1.5 expression, no difference between cardiomyocytes with and without co-culture was detectable in whole cell lysates (Fig. 3d). Since overall protein expression of Na_v_1.5 in cardiomyocytes was identical with and without coculture and given the role of Cx43 in regulating membrane recruitment of Na_v_1.5, we performed proximity ligation assay (PLA) to evaluate a possible direct interaction of Na_v_1.5 and Cx43 (Fig. 3e). Macrophage co-culture induced a 1.62 fold increase in Na_v_1.5/Cx43 complexes in cardiomyocytes in vitro. To translate our in vitro findings into the in vivo model, Na_v_1.5 protein expression was analyzed in whole heart lysates of MM^DTR^ and control mice. As depicted in Fig. 3f no difference was evident. However, performing PLA with either detecting the pairs Na_v_1.5 with Cx43 or Na_v_1.5 with syntrophin, a marker protein of the cardiomyocyte`s lateral cell membrane, both measures confirmed reduced expression of Na_v_1.5 in the macrophage-depleted mouse hearts (Fig. 3g and Suppl. Figure 2f). Taken together, these data indicate that macrophages support the effects of flecainide by regulating membrane recruitment of Na_v_1.5 through Cx43 in cardiomyocytes in vitro and in vivo.

## Discussion

Based on the seminal work of *Hulsmans *et al. and others, the field of electro-immunology has gained exciting momentum in recent years. A pivotal role to modulate AV-conduction, arrhythmia, and sudden cardiac death is attributed to cardiac macrophages [8, 10, 13, 14, 21, 23]. Our here presented data extends these insights regarding AV-conduction and provides an additional mechanisms of cardiac macrophages affecting anti-arrhythmic therapy.

To our knowledge, it is the first time that the LysM^Cre^xCsf1R^LsL−DTR^ (MM^DTR^) was characterized with focus on cardiac electrical conduction. The MM^DTR^ model holds the advantage, that injection of DTx selectively depletes monocytes and macrophages [20]. With this specific depletion method, we did not observe any AV-conduction disturbances upon monocyte and macrophage removal. To put further stress on the conduction system and to demask a potential vulnerability for an AV-node delay, we administered the class IC antiarrhythmic drug flecainide to MM^DTR^ mice [6, 25]. Even under these conditions, we were not able to find any disturbances upon electrical cardiac conduction or a higher AV-node vulnerability. Our results indicate that macrophages/monocytes are not essential for maintaining cardiac electrical conduction. Previously reported effects on electrical cardiac conduction are highly variable within different myeloid depletion model systems: Knockout of Cx43 in fraktalkine receptor positive macrophages (Cx3cr1 Cx43^−/−^ mice) [13] and congenital macrophage depletion (Csf1op mice) lead to a 1st degree AV-block [13]. Clodronate-mediated macrophage depletion resulted in no AV-node conduction abnormality [13, 23], whereas only after depletion of all myeloid cells (CD11b^DTR^ model) a real 3rd degree AV-block occurs [13]. Possibly, the described varying phenotypes on electrical cardiac conduction are explainable by compensatory mechanisms of other subsets or depletion of a broader range of myeloid cell subsets. In contrast to the aforementioned mouse models [13, 23], our MM^DTR^ model which targets only macrophages/monocytes clearly shows that the macrophage/monocyte subset is not essential in maintaining AV-conduction in mice.

Beside the analysis of AV-conduction, we were able to detect a new possible mechanism for how macrophages enhance the effects of anti-arrhythmic drugs in cardiomyocytes. Flecainide is an anti-arrhythmic drug with high affinity to sodium channels –and in particular blocks Na_v_1.5. Interestingly, we found significantly higher heart rates remained in MM^DTR^ mice compared to controls after the application of flecainide, suggesting that depletion of macrophages in the murine myocardium leads to an impaired effect of this antiarrhythmic drug. We show close proximity of Cx43 and Na_v_1.5 between macrophages and cardiomyocytes, in vivo as well as in vitro. Our data suggest that the interaction of macrophages and cardiomyocytes induces an enhanced surface expression of Na_v_1.5. Furthermore, we were able to detect a functional coupling of macrophages to cardiomyocytes via gap junctions in vitro, which was the basis for our implemented cell culture model for further patch-clamp measurements. Electrotonic coupling of macrophages to cardiomyocytes via Cx43 is well described [13, 21]. Our patch-clamp data corroborated an increased depolarization and show a significantly prolonged APD50 in the presence of macrophages under basal conditions as described previously [13]. These findings are in line with patch-clamp analyses from *Fei *et al*.*, who showed that coupling of M1-polarized macrophages resulted in significantly increased APD50 and APD90 [8]. Furthermore, our data show that macrophages significantly enhance V_max_ even at a more depolarized RMP. V_max_ is significantly influenced by the density of sodium channels at the cell membrane (C) and neighboring macrophages induce a higher surface expression of Na_v_1.5, possibly through the Cx43-Na_v_1.5 interaction.

Thus, V_max_ is of higher magnitude, but in addition conduction velocity in the tissue may be increased as well, both caused by a higher total amount of Na_v_1.5 channels but also Cx43, which are known to closely interact in a molecular complex [22]. Administration of flecainide resulted in a pronounced reduction of Vmax, APD50 and APD90 only in cardiomyocytes coupled to macrophages compared to controls. An additional functional uncoupling of gap junctions between macrophages and cardiomyocytes through flecainide can not be completely excluded. However, our *in vitr*o and in vivo data suggest that the higher effects of flecainide on cardiomyocytes in the presence of macrophages result from the higher surface expression of Na_v_1.5.

The exact mechanism of how overexpression of Cx43 through macrophages leads to higher surface expression of Na_v_1.5 on the cardiomyocyte cell membrane is unresolved yet. A recent study identified amphiregulin (AREG) as a mediator in macrophages which maintains electrophysiological stability [23]. It must be stated, that in our experimental protocol potential effects of secretory mediators like AREG or cytokines could be excluded through treatment of the control group with macrophages’ supernatant. Coupling of macrophages to cardiomyocytes via Cx43 seems not to be exclusive for the AV-node, as Cx43 was detected in almost all isolated ventricular macrophages [21]. The concomitant Cx43—Na_v_1.5 interaction and their translational relevance was previously shown in a study which lead to a significant loss of Na_v_1.5 and severe ventricular arrhythmia [18] after deletion of the last five amino acids of Cx43 – representing a binding motif for tight-junction-protein-1. *Fei *et al*.* could show the formation of gap junctions between cardiomyocytes and pro-inflammatory macrophages in myocardial infarction (MI) border zones, that are associated with ventricular arrhythmia in vivo [8]. Interestingly, ischemia through MI contributes additionally to electrical uncoupling via induction of dephosphorylation of ventricular Cx43 protein, which normally exists in a phosphorylated state [3]. These findings demonstrate the impact of macrophages on heart rhythm disorders and stretches further as indicated by recent work in atrial fibrillation and ischemia-induced ventricular arrhythmias [1, 10, 14, 19, 23, 24].

Taken together, our findings show that cardiac resident macrophages are critical modulators of flecainide effects. We did not observe conduction abnormalities after the depletion of macrophages by removing Csf1r and LysM double-positive cells in mice. Mechanistically, we propose that the presence of macrophages in the heart leads to higher effects of flecainide on cardiomyocytes via Cx43-dependent enhanced recruitment of Na_v_1.5 to the cell membrane. This may be a reason for the excess of death after myocardial infarction (MI) in patients under flecainide treatment observed in the Cardiac Arrhythmia Suppression Trial (CAST) [5], as MI promotes cardiac inflammation with well-documented changes in macrophages’ polarity and quantity [4, 12, 15]. A greater understanding of the pharmacological influence on cardiac immune cells and vice-versa, may improve future treatment approaches and help to protect patients from adverse drug effects.

## Supplementary Information

Below is the link to the electronic supplementary material.

Supplementary file1 (JPG 1877 KB)

Supplementary file2 (JPG 5513 KB)

Supplementary file3 (Docx 23 KB)
