# Supplementary figures and images for: Macrophages enhance sodium channel expression in cardiomyocytes
Source: Basic Res Cardiol. 2024 Oct 9;119(6):1063–73. doi: 10.1007/s00395-024-01084-8 (PMC11628573; doi:10.1007/s00395-024-01084-8)

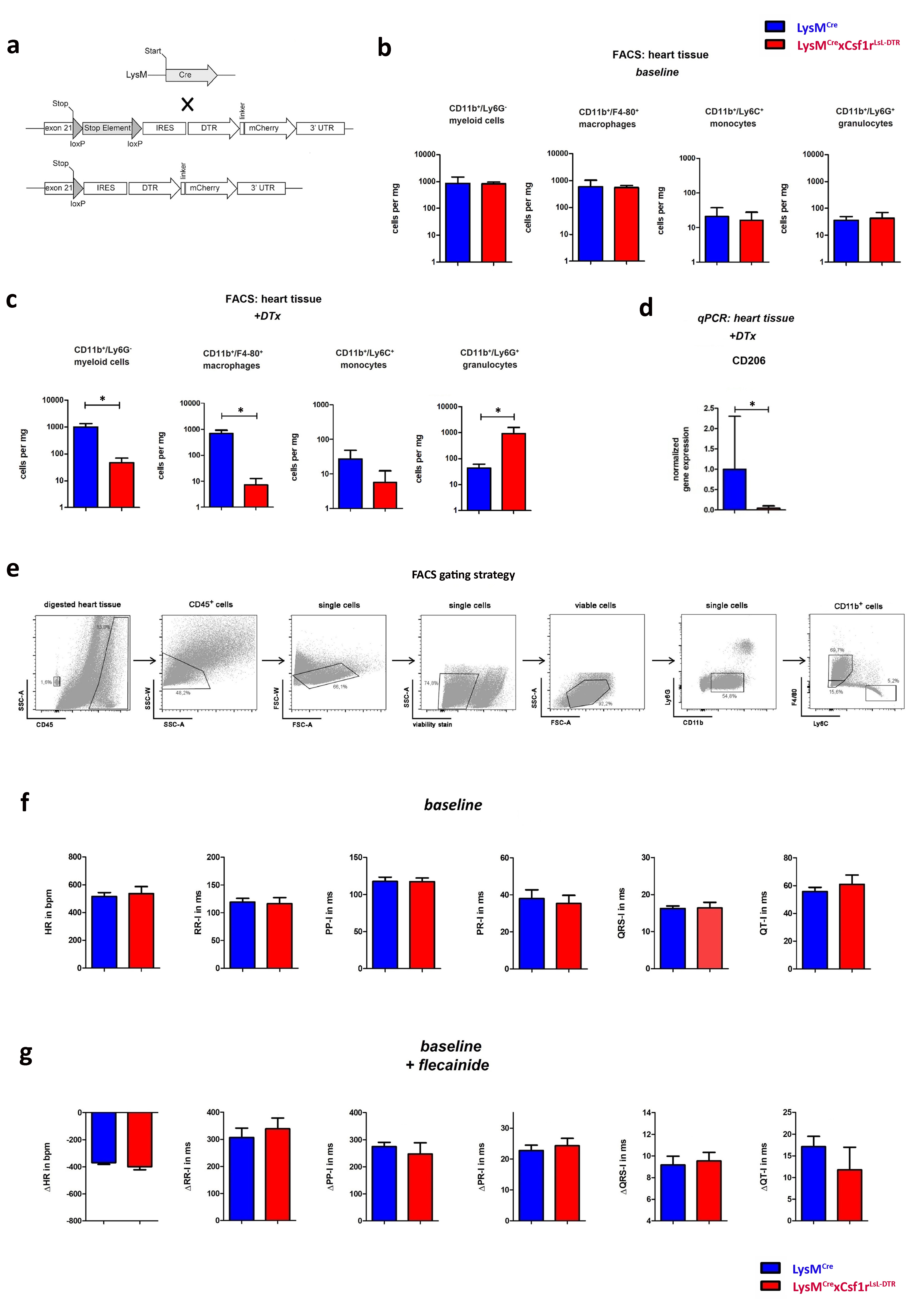

Supplement: Supplementary file 1 — Supplementary file1 (JPG 1877 KB) [file 395_2024_1084_MOESM1_ESM.jpg]

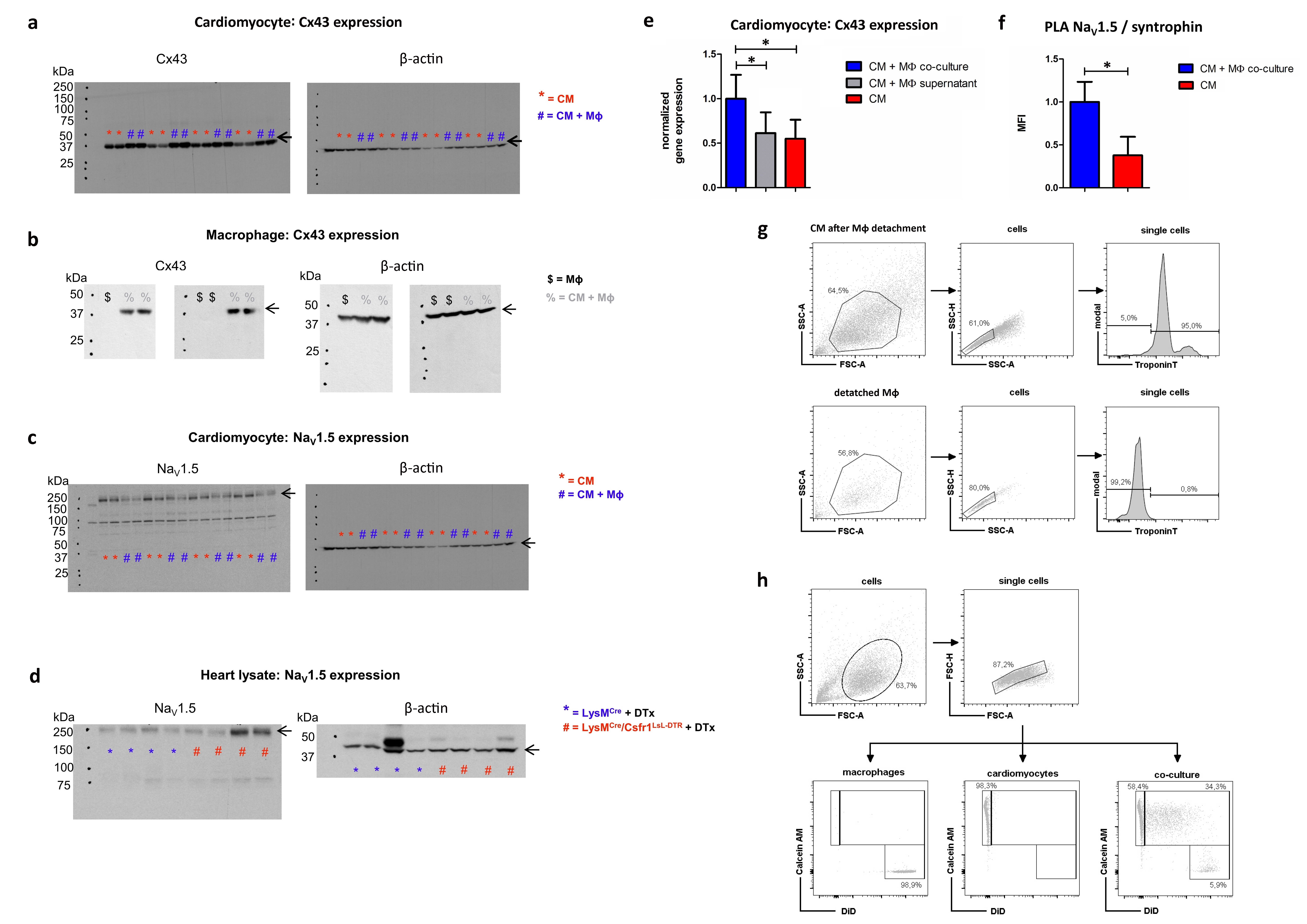

Supplement: Supplementary file 2 — Supplementary file2 (JPG 5513 KB) [file 395_2024_1084_MOESM2_ESM.jpg]
