# Supplementary material for: Macrophages enhance sodium channel expression in cardiomyocytes
Source: Basic Res Cardiol. 2024 Oct 9;119(6):1063–73. doi: 10.1007/s00395-024-01084-8 (PMC11628573; doi:10.1007/s00395-024-01084-8)
Supplement: Supplementary file 3 — Supplementary file3 (Docx 23 KB) [file 395_2024_1084_MOESM3_ESM.docx]

**Suppl. Figure 1:**

**(a)** Genetic background of the LysM^Cre^ x Csf1r^LsL-DTR^ (MM^DTR^) mouse as described by Schreiber et al. [20]. **(b)** Comparison of quantity of myeloid cells, macrophages, monocytes and granulocytes between LysM^Cre^ mice (control group, n=4) and LysM^Cre^xCsf1r^LsL-DTR^ mice (n=4) shows no significant differences without intraperitoneal application of DTx. **(c)** Comparison between LysM^Cre^ mice (control group, n=4) and LysM^Cre^xCsf1r^LsL-DTR^ mice (n=4) shows a significant decrease of macrophages in LysM^Cre^ x Csf1r^LsL-DTR^ mice after intraperitoneal application of DTx confirming successful depletion conditions. **(d)** Application of DTx in LysM^Cre^xCsf1r^LsL-DTR^ mice (n=4) leads to a sufficient depletion of CD206^+^ M2-polarized macrophages, compared to LysM^Cre^ mice (n=4). **(e)** FACS gating strategy for murine cardiac macrophages. Leukocytes were identified as CD45^+^ cells, followed by forward/sideward scatter to identify single cells. After viability staining, focus was set on Ly6G^-^ and CD11b^+^ cells. Finally, cardiac macrophages were identified as F4/80^+^/Ly6C^-^ cells. **(f/g)** Comparison of ECG values between LysM^Cre^ mice (control group, n=4) and LysM^Cre^ x Csf1r^LsL-DTR^ mice (n=4) shows no significant differences in quantity before or after application of DTx**.**

**Suppl. Figure 2:**

**(a)** Full immunoblot images of Cx43 and beta-actin (β-actin) expression in CMs (n=8); CM+Mφ (n=8) and **(b)** macrophages (Mφ n=1, CM+Mφ n=2) after co-culture experiments. **(c)** Full immunoblot images of Na_v_1.5 and β-actin expression in cardiomyocytes after co-culture experiments (CM n=8, CM+Mφ n=8). **(d)** Full immunoblot images of Na_v_1.5 and β-actin expression in heart lysates (n=4). **(e)** Gene expression of Cx43 in cardiomyocytes after incubation with macrophage supernatant or direct co-culture condition (n=6). Only direct cell contact induced Cx43 in CMs. **(f)** PLA between Na_v_1.5 and syntrophin shows a significant decrease in LysM^Cre^xCsf1r^LsL-DTR^ mice (red column, n=4) compared to LysM^Cre^ mice (control group, blue column, n=4)**. (g)** FACS analysis shows an effective detachment of macrophages from cardiomyocytes in our *in vitro* model with minimal cross-contamination. **(h)** Cardiomyocytes and macrophages were labeled separately with either Calcein oder DiD (Cell Meter™ Fluorescence Gap Junction Tracing Kit). Co-culture conditions leads to double positive cells indicating a functional coupling of cardiomyocytes and macrophages through gap junctions after 24h. *p<0.05, data are shown as mean ± SD.

**Supplemental material**

**Antibodies and reagents**

| **name** | **clone** | **supplier** | **dilution** |
| --- | --- | --- | --- |
| anti-CD45 | Clone 30-F11 | BD Bioscience | 1:50 |
| FVS | - | BD Bioscience | 1:1000 |
| Fc-block CD16/CD32 | 2.4G2 | BD Bioscience | 1:100 |
| anti-CD11b | M1/70 | BD Bioscience | 1:50 |
| anti-Ly6G | 1A8 | BD Bioscience | 1:100 |
| anti-Ly6C | AL-21 | BD Bioscience | 1:50 |
| anti-Na_v_1.5 | polyclonal | Alomone | 1:200 |
| anti-connexin43 | polyclonal | Sigma-Aldrich | 1:1000 |
| anti-connexin43 | CX-1B1 | Invitrogen | 1:100 |
| anti-pan-syntrophin | 1351 | Invitrogen | 1:100 |
| Rat anti-mouse F4/80 | A3-1 | Bio-Rad | 1:150 |
| Biotin Goat Anti-Rat Ig | polyclonal | BD Bioscience | 1:50 |

**Primer sequences**

|  | **forward** | **reverse** |
| --- | --- | --- |
| F4/80 ms | TCACCTTGTGGTCCTAACTCAG | TCAGACACTCATCAACATCTGCG |
| CD206 ms | TGGAACCACCACTGACTACG | TCTCGCTTCCCTCAAAGTGC |
| CXCL10 ms | CCACGTGTTGAGATCATTGCC | TCACTCCAGTTAAGGAGCCC |
| Cx43 ms | TCCTTTGACTTCAGCCTCCAAG | TGGGCACCTCTCTTTCACTTAAT |
| Na_v_1.5 ms | CGTCATGGCATACACAACTGAA | TGACCGATATAGTTTTCAGGGC |
| Cx43 rt | CATTAAGTGAAAGAGAGGTGCCC | GGTGGAGTAGGCTTGGACCT |
| Na_v_1.5 rt | ATTCACCTTCCTTCGGGACC | AAATTCAGTTGTGTATGCCATGA |
